# The effect of inheritance of IgE responsiveness on the susceptibility of mice to *Trichinella spiralis* infection

**DOI:** 10.3389/fimmu.2023.1185094

**Published:** 2023-05-19

**Authors:** Naohiro Watanabe

**Affiliations:** Department of Tropical Medicine, The Jikei University School of Medicine, Tokyo, Japan

**Keywords:** IgE, *Trichinella spiralis*, susceptibility, genetic control, atopy, mouse

## Abstract

IgE antibodies are likely involved in host protection. *Trichinella spiralis* is a helminth that induces protection through IgE antibodies. The present study examined *T. spiralis* susceptibility in high and low IgE responder mice, with a specific focus on the inheritance of IgE responsiveness, which controls IgE production specific for the IgE isotype and non-specific for antigens. Furthermore, low IgE responsiveness is inherited as a recessive trait under a single gene, which is not linked to the H-2 gene. This study determined the total IgE and anti-*T. spiralis* IgE antibody levels after *T. spiralis* infection in low IgE responder SJL/J mice were several times lower than those in high IgE responders, such as the BALB/c mice. An IgE-dependent susceptibility to *T. spiralis*, evaluated in mice treated with anti-IgE antibodies and in control mice, was observed in high IgE responder mice but not in low IgE responder mice. The inheritance of IgE responsiveness and susceptibility to *T. spiralis* was investigated using crosses of SJL/J with high IgE responders. All of the (BALB/c × SJL/J) F1 and half of the (BALB/c × SJL/J) F1 × SJL backcross progenies were high IgE responders after *T. spiralis* infection. Total IgE and antigen-specific IgE antibody levels were correlated and not linked to H-2. It should be noted that high IgE responders always exhibited low susceptibility, suggesting that the trait of IgE responsiveness functions as a trait of susceptibility to *T. spiralis*.

## 1 Introduction

Previous studies have revealed that differences in IgE responsiveness were found in mice, namely high IgE responder BALB/c mice and low IgE responder SJL/J mice (1). I have previously reported the mechanism of the low IgE response in SJL/J mice. Although helper T and B cells, required for IgE production, were not impaired in SJL/J mice (2, 3), IgE production was rather actively suppressed by T cells (4–6) This study has found evidence that CD4 T cells suppress IgE production specific for an IgE isotype regardless of immunized antigens (4). Watanabe et al. (1977) were the first to report that T cells with suppressive function express CD4 on the cell surface (7). Furthermore, the inheritance of the low IgE response in SJL/J mice was analyzed by making crosses with high IgE responder BALB/c mice. The (BALB/c x SJL/J) F1 mouse was the high IgE responder, and (F1 x SJL/J) backcross mice were separated into high and low IgE responders at a 1:1 ratio. These results indicate that the characteristic of an SJL/J mouse being a low IgE responder is inherited as a recessive trait controlled by a single gene. In addition, this trait is not linked to the H–2 gene complex (4). According to the features of IgE–specific and antigen–nonspecific control, the trait regulates the total IgE level. In humans, individuals with atopy show high levels of total IgE and frequently produce IgE antibodies to multiple allergens.

Helminth infections are characteristically associated with type 2 immune responses, including T helper type 2 (Th2) cells and type 2 innate lymphoid cells, resulting in IgE production, eosinophilia, and intestinal mastocytosis. The type 2 immune response has been considered to be responsible for protection against helminths in a complex manner (8, 9). Although IgE antibodies are a feature of type 2 immune response, their protective role against helminth infection remains ill–defined.

*Trichinella spiralis* is a representative helminth showing IgE–dependent protection in rodent hosts (10). Rats with suppression of IgE antibodies are more susceptible to primary infection with *T. spiralis* (11), which is the first report that IgE antibodies are critical for protection against helminths *in vivo*. Both IgE antibodies and CD4 T cells mediate the rapid expulsion of *T. spiralis* in rats (12, 13). If rats have been immunized, the nematode is quickly expelled from the intestine after oral challenge infection with muscle larvae. Susceptibility to *T. spiralis* is increased in IgE–deficient BALB/c mice following IgE gene knockout. In IgE–knockout mice, the number of muscle larvae is twice that in IgE–producing control BALB/c mice (14). Contrasting results have been obtained in congenitally IgE–deficient SJA/9 mice and IgE–producing SJL/J mice with background genes of SJA/9 mice. Moreover, the numbers of muscle larvae after primary and secondary infection in these mice did not differ significantly (15). The discrepancy in these results in mice might be explained by their genetic background. Genetic regulation of susceptibility to *T. spiralis* has been reviewed elsewhere (16, 17).

This study examined the susceptibility to *T. spiralis* infection in high and low IgE responder mice based on a previous study (4), and the relationship between susceptibility and inheritance of IgE responsiveness.

## 2 Materials and methods

### 2.1 Reagents

Pepsin (Nacalai Tesque Co., Kyoto, Japan), bovine serum albumin (BSA) (Sigma–Aldrich, St. Louis, MO, USA), Evans blue (Sigma–Aldrich), streptavidin–peroxydase (Zymed Laboratories, Inc., San Francisco, CA, USA), azino–bis (3–ethylbenz–thiazoline–6–sulfonic acid) (Sigma–Aldrich), and H_2_O_2_ (Mitsubishi Gas Chemical Co., Tokyo, Japan) were purchased from the indicated sources.

### 2.2 Antigens

The *T. spiralis* antigen was a soluble extract of muscle larvae of *T. spiralis*. The larvae obtained from mice infected with 400 *T. spiralis* larvae for 2 months were homogenized and dialyzed against borate–buffered saline (pH 8.0) for 1 day. The antigen was a supernatant obtained after centrifugation at 10,000 rpm for 30 minutes at 4˚C (15). Dinitrophenyl (DNP)–conjugated *T. spiralis* antigen or DNP–BSA was prepared as previously described (18).

### 2.3 Antibodies

Rat anti–mouse IgE monoclonal antibodies (6HD5, HMK–12, P2B10) were derived from hybridomas established by us, and biotinylated anti–mouse IgE monoclonal antibody (HMK–12) was prepared (19). An anti–DNP IgE monoclonal antibody (SPE–7) was purchased from Seikagaku Kogyo (Tokyo, Japan). Fluorescein– conjugated anti–H–2^d^ was purchased from PharMingen (San Diego, CA, USA.).

### 2.4 Animals

The BALB/c, C3H/He, and (C57Bl/6 × DBA/2) F1 mice were purchased from Japan SLC (Hamamatsu, Japan). The SJL/J mice were purchased from the Ohmura Institute for Laboratory Animals (Kanagawa, Japan). The ASW mice were purchased from The Jackson Laboratory (Bar Harbor, ME, USA), and (ASW × SJL/J) F1, (BALB/c × SJL/J) F1, and (BALB/c × SJL/J) F1 ×SJL/J backcross mice were bred and maintained in accordance with the institutional guidelines at the Animal Facility of The Jikei University. IgE responsiveness was determined according to a previous study (4). SJL/J mice are low IgE responders while other strains are high IgE responders. Wistar rats for passive cutaneous anaphylaxis (PCA) reactions were purchased from Ishikawa Laboratory Animals (Saitama, Japan). Selective IgE–suppressed mice were produced through repeated injections of anti–IgE monoclonal antibodies, per our previous report (20). Pregnant mice were intravenously injected with 2 mg of rat IgG2a 7 days before delivery. Neonatal mice were given an intraperitoneal injection of 5 μg of purified anti–mouse IgE monoclonal antibody (6HD5, rat IgG2a) within 24 hours of birth. The mice were injected with 5 μg of anti–IgE (Days 0 and 3), followed by 10 μg of anti–IgE (Days 6, 9, and 12), 10 μg of anti–IgE (Days 16, 20, 24), and thereafter 30 μg anti–IgE every 7 days until the end of the experiments. Control mice were injected with normal rat IgG2a according to the same schedule as anti–IgE treatment. This method prevented antibody–induced anaphylactoid shock due to rat IgG2a. Importantly, no mice died during the anti–IgE treatment. Anti–rat IgG2a antibodies were not detected in treated mice between 6 and 10 weeks of age (20). Experiments of anti–IgE treated mice were performed separately for each strain. All experiments were approved by the Animal Care and Use Committee of The Jikei University (No.21–013C1 and 25–073).

### 2.5 Infection, worm recovery, and immunization

Mice were inoculated orally with 100 or 200 muscle larvae of *T. spiralis*. As we have previously reported, 100 muscle larvae were used to infect mice in experiments of anti–IgE treatment (15). To induce a strong carrier effect for DNP*–T. spiralis* antigen immunization, 200 muscle larvae were used to infect mice in crossing experiments (4). High levels of ant–DNP IgE production were required for the evaluation of IgE responsiveness in each mouse. Larval recovery was determined 4 – 8 weeks after infection by digestion of the eviscerated carcasses of the infected mouse. Larval recovery was performed 4 – 5 weeks after infection in experiments of anti–IgE treatment without immunization. Larval recovery was performed 8 weeks after infection in the crossing experiments in which mice were immunized with DNP–*T. spiralis* antigen 4 weeks after infection to determine IgE responsiveness with anti–DNP IgE PCA titer 1 – 3 weeks after immunization. The tissue was digested in 200 mL of 0.5% pepsin–HCl at 37˚C for 3 – 4 hours until no muscle tissue remained. The larvae were collected *via* sedimentation and washed with saline and then finally resuspended in 5mL of saline. The number of muscle larvae in five samples of 20 μL of worm suspension was counted under a microscope, and the total larval count in each mouse was calculated (15). In crossing experiments, mice were intraperitoneally immunized with 10 μg DNP–*T. spiralis* antigen with 2 mg of Al (OH)_3_ 4 weeks after infection to identify IgE responsiveness in each mouse by anti–DNP IgE antibody production 1 – 3 weeks after immunization. The IgE responsiveness of each backcross mouse was also examined by anti–*T. spiralis* IgE antibody production 3 weeks after infection. In backcross mice experiments, larval recovery was performed 8 weeks after infection to confirm the IgE responsiveness by anti–DNP IgE production and anti–*T. spiralis* IgE production.

### 2.6 Study design

In experiments of anti–IgE treatment, mice were treated with anti–IgE or control IgG before and during experiments. Mice (five per group) were infected with 100 *T. spiralis* muscle larvae. Total IgE levels in the plasma were measured 2 – 4 weeks after infection. Mice were sacrificed 4 – 5 weeks after infection for *T. spiralis* larval recovery.

In backcross mice experiments, 24 or 18 backcross mice were infected with 200 *T. spiralis* muscle larvae. Total IgE levels in the plasma were measured 2 – 4 weeks after infection. Anti–*T. spiralis* IgE antibody was examined 3 weeks after infection. To determine IgE responsiveness in each backcross mouse, mice were immunized with DNP–*T. spiralis* antigen 4 weeks after infection. Anti–DNP IgE levels were examined 1 – 3 weeks after DNP–*T. spiralis* antigen immunization. Mice were sacrificed 8 weeks after infection (4 weeks after immunization) for *T. spiralis* larval recovery.

In FI experiments, (BALB/c × SJL/J) F1. BALB/c and SJL/J mice (five per group) were infected with 200 *T. spiralis* muscle larvae. Mice were immunized with DNP–*T. spiralis* antigen 4 weeks after infection. Anti–DNP IgE antibodies were measured 1 – 3 weeks after immunization. Mice were sacrificed 8 weeks after infection (4 weeks after immunization) for *T. spiralis* larval recovery. Next, (ASW × SJL/J) F1, ASW, and SJL/J mice (five per group) were infected with 200 *T. spiralis* muscle larvae. Anti–*T. spiralis* IgE antibody was examined 3 weeks after infection. Mice were immunized with DNP–*T. spiralis* antigen 4 weeks after infection to confirm IgE responsiveness 1 and 2 weeks after immunization. Mice were sacrificed 6 weeks after infection (2 weeks after immunization) for *T. spiralis* larval recovery.

### 2.7 Enzyme–linked immunosorbent assay

The levels of total IgE in the plasma were determined with ELISA using anti–mouse IgE monoclonal antibodies (19). Microtiter plates (Immulon 2, Thermo Fisher Scientific, Inc., Waltham, MA, USA) were coated with an anti–mouse IgE monoclonal antibody (6HD5) at room temperature for 1 hour and blocked with 1% BSA for 1 hour. After the application of plasma samples and standards (anti–DNP IgE monoclonal antibody SPE–7) for 1 hour, a biotinylated anti–mouse IgE monoclonal antibody (HMK–12) was added for 30 minutes followed by the addition of streptavidin–peroxidase. The plates were washed between steps with phosphate–buffered saline containing–0.5% (v/v) Tween 20. The substrate solution (azino–bis (3–ethylbenz–thiazoline–6–sulfonic acid) and H_2_O_2_) was added, and the reaction was stopped with citrate and was read at 490 nm with an ELISA plate reader. In the experiments of anti–IgE treated mice, microtiter plates were coated with an anti–mouse IgE monoclonal antibody (P2B10). These anti–mouse IgE monoclonal antibodies recognized different epitopes of IgE molecules (19).

### 2.8 Passive cutaneous anaphylaxis reaction

Antigen–specific IgE antibodies were detected through PCA reactions in rats (21).

Serial dilutions of plasma samples (0.1ml aliquots) were injected intradermally into the shaved dorsal skin of Wistar rats. After a 24–hour–sensitization period, 1 mg of *T. spiralis* antigen for anti–*T. spiralis* IgE antibody or DNP–BSA for anti–DNP IgE antibody was intravenously injected with 1 mL of 0.5% Evans blue. The reactions were examined 30 minutes after the challenge injection. The titer is expressed as the highest dilution. In backcross mice experiments, mice with an anti–DNP IgE PCA titer higher than 160 were classified as high responders, and mice with titers lower than 40 were classified as low responders. Mice with an anti–*T. spiralis* IgE PCA titers above 80 and below 40 were categorized as high and low responders, respectively.

### 2.9 Flow cytometric analysis

White blood cells in peripheral blood from (SJL/J ×BALB/c) F1 × SJL/J backcross mice were stained with fluorescein–conjugated anti–H–2^d^ antibody and analyzed using a flow cytometer cell sorter (FACStar, Becton Dickinson and Company, Franklin Lakes NJ, USA).

### 2.10 Statistical analysis

Statistical analysis was performed with an unpaired Student’s *t*–test using R statistical software version 4.2.3 (R Project for Statistical Computing). A p–value< 0.05 was considered to be statistically significant.

## 3 Results

### 3.1 IgE production in high and low IgE responders after *T. spiralis* infection

**Figure 1:**
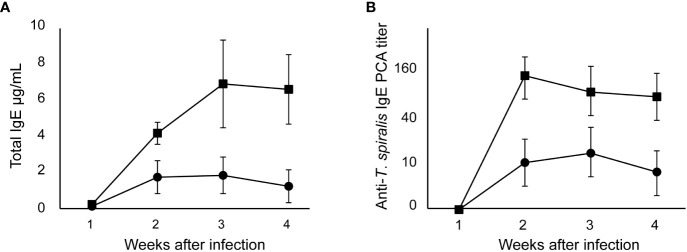
IgE production after *T. spiralis* infection in high and low IgE responder mice. High IgE responder BALB/c mice (●) and low IgE responder SJL/J mice (■)were infected with 100 *T. spiralis* muscle larvae. **(A)** Total IgE levels. **(B)** Anti–*T. spiralis* IgE antibody determined by PCA reaction. The mean ± SD is shown. Five mice per group. * P<0.01.

Production of IgE by high responder BALB/c mice was detected 2 weeks after oral infection of 100 muscle larvae of *T. spiralis* and peaked 3 weeks after. From an IgE level of 0.1 μg/mL in an uninfected mouse, total IgE levels in infected BALB/c mice increased by 40 – 60 times (**Figure 1A**). The kinetics of IgE production in SJL/J mice was similar to that in BALB/c mice. However, the total IgE levels in low IgE responder SJL/J mice were significantly lower than those in BALB/c mice. In both strains of mice, anti–*T. spiralis* IgE antibodies were detected 2 weeks after infection and persisted thereafter (**Figure 1B**). The anti–*T. spiralis* IgE PCA titers in BALB/c mice were 10 times as high as those in SJL/J mice.

### 3.2 The effect of anti–IgE treatment on susceptibility to *T. spiralis* in high and low IgE responders

Repeated injections of anti–IgE antibodies into mice resulted in marked decreases of total IgE levels to undetectable levels with our ELISA system (<20 ng/mL) in the plasma of all strains of mice (**Table 1**). The amount of IgE produced was lower in low IgE responder SJL/J mice than in high IgE responder mouse strains. The ASW mice, which have the same *H–2^s^* haplotype as SJL/J mice, were high IgE responders.

**Table 1:** The effect of anti–IgE treatment on susceptibility to *T. spiralis* in high and low IgE responders.

Mouse^1^		IgE response	Total IgE (μg/ml) ^2)^	*T. spiralis* muscle larvae Recovered^3)^ (% reduction)	P
BALB/c	Control	High	7.6 ± 2.4	6,700 ± 887 (48%)	
	Anti–IgE	Non	0	12,800 ± 3,178	0.028
C3H/He	Control	High	4.6 ± 0.9	10,175 ± 1,272 (19%)	
	Anti–IgE	Non	0	12,638 ± 1,695	0.054
BDF1	Control	High	7.0 ± 1.3	4,200 ± 1195 (40%)	
	Anti–IgE	Non	0	6,954 ± 698	0.0013
ASW	Control	High	7.6 ± 0.5	8,433 ± 1,498 (28%)	
	Anti–IgE	Non	0	11,771 ± 1,606	0.0026
SJL/J	Control	Low	1.5 ± 0.9	9,540 ± 1453 (–15%)	
	Anti–IgE	Non	0	8,300 ± 884	0.14

^1)^Five mice per group.

^2)^Mean total IgE levels in 2 – 4 weeks after infection. Mean ± SD.

^3)^T. spiralis muscle larvae were recovered 4 – 5 weeks after infection with 100 muscle larvae. Mean ± SD.

In BALB/c mice treated with anti–IgE antibody, the number of muscle larvae 4 – 5 weeks after infection was significantly greater than in control mice (**Table 1**). Similar results were obtained in other high IgE responders such as BDF1 and ASW mice but not in C3H/He mice. Contrary to high IgE responders, larval counts did not differ significantly in low IgE responder SJL/J mice compared to control mice following anti–IgE antibody treatment.

### 3.3 Inheritance of susceptibility to *T. spiralis* in (high IgE × low IgE) F1 mice

**Figure 2:**
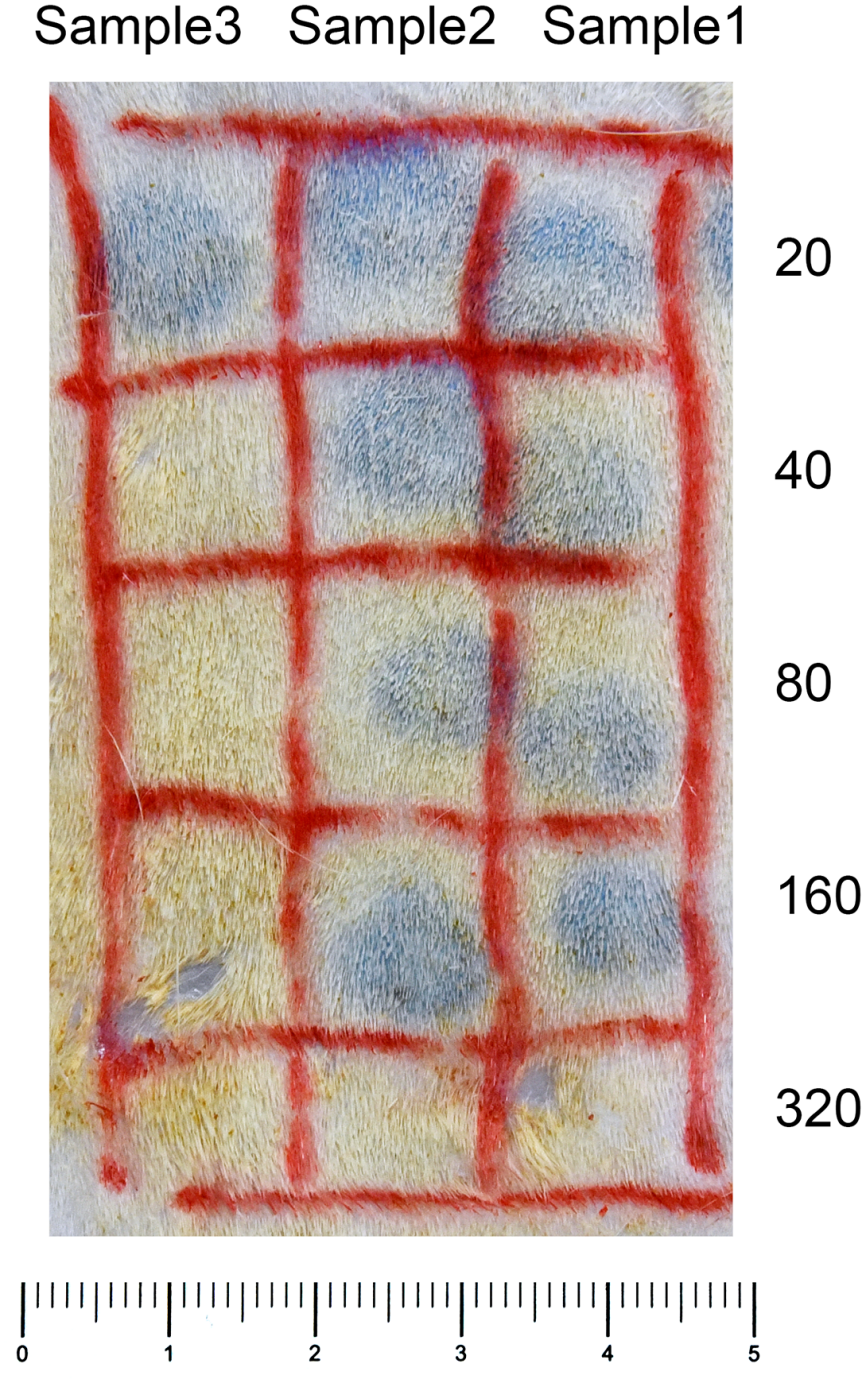
Anti–DNP IgE PCA reaction. Serial dilutions of plasma samples (0.1mL aliquots) were injected intradermally into the skin of rats. After a 24–hour–sensitization period, 1 mg of DNP–BSA was intravenously injected with 1 mL of 0.5% Evans blue. The reactions were examined 30 minutes after the challenge injection. Anti–DNP IgE PCA titers of Samples 1 and 2 were 160, and Sample 3 was 20.

**Figure 3:**
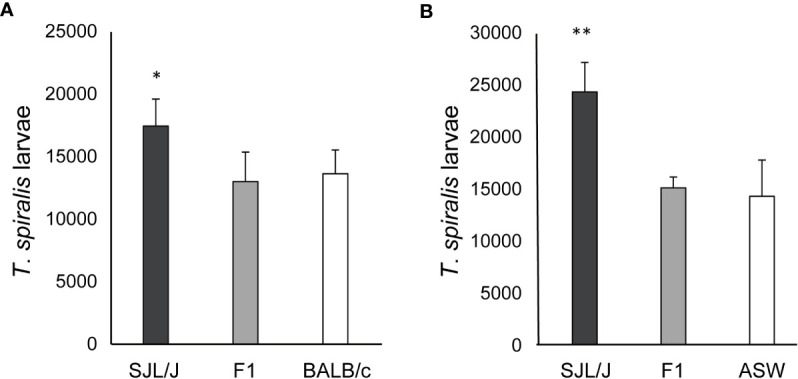
Susceptibility to *T. spiralis* in (high IgE × low IgE) F1 mice. **(A)** (BALB/c × SJL/J) F1, BALB/c, and SJL/J mice were infected with 200 muscle larvae of *T. spiralis*. Muscle larvae were counted 8 weeks after infection. **(B)** (ASW × SJL/J) F1, ASW, and SJL/J mice were infected with 200 muscle larvae of *T. spiralis*. Muscle larvae were counted 6 weeks after infection. The mean ± SD is shown. Five mice per group. * P<0.05, ** P<0.01 .

F1 and parental mice were immunized with DNP–*T. spiralis* antigen 4 weeks after infection with 200 *T. spiralis* muscle larvae. In all individual BALB/c and (BALB/c × SJL/J) F1 progeny mice, anti–DNP IgE PCA titers were 160 after immunization. Titers in all parental SJL/J mice were 20 (**Figure 2**), thereby, indicating that the low IgE trait is recessive. The number of larvae in F1 progeny mice 8 weeks after infection was similar to parental BALB/c mice and was significantly lower than in SJL/J mice (**Figure 3A**). Low IgE response resulted in high susceptibility to *T. spiralis*.

Six weeks after infection with 200 *T. spiralis* muscle larvae, the larval counts were significantly lower in (ASW × SJL/J) F1 mice and ASW mice than in SJL/J mice (**Figure 3B**) despite these strains having the same *H–2^s^* haplotype. IgE responsiveness of F1 and parental mice was detected by anti–*T. spiralis* IgE PCA titers 3 weeks after infection. Anti–*T spiralis* IgE PCA titers were 80 in (ASW × SJL/J) F1 mice and ASW mice and 20 in SJL/J mice. These data confirm that the low IgE trait is recessive. Moreover, low IgE responses again resulted in high susceptibility to *T. spiralis*.

### 3.4 Inheritance of susceptibility to *T. spiralis* in (BALB/c × SJL/J) F1 × SJL/J backcross mice

(BALB/c × SJL/J) F1 × SJL/J backcross mice were classified as high and low IgE responders by anti–DNP IgE PCA titer 1 – 3 weeks after DNP–*T spiralis* antigen immunization. This work has identified 11 high IgE responders and 13 low IgE responder mice. Total IgE levels in backcross mice classified as high IgE responders, owing to anti–DNP IgE PCA titers >160, were approximately twice as high as those in low IgE responder mice, with anti–DNP IgE PCA titers <40 (**Table 2**). The muscle larva count 8 weeks after infection with 200 *T. spiralis* muscle larvae was significantly higher in low IgE responders than in high IgE responders. Similar results were obtained from three separate experiments.

**Table 2:** Susceptibility to *T. spiralis* in (BALB/c **×** SJL/J) F1 × SJL/J backcross mice classified by anti–DNP IgE production and H–2 haplotype.

Backcross mouse (n)	Total IgE^1)^ (μg/ml)	*T. spiralis* muscle larvae recovered^2)^	P^3)^
High IgE responder (11) ^4)^	13.3 ± 3.0	12,081 ± 2,050	
Low IgE responder (13)	6.9 ± 1.6	18,705 ± 4,832	<0.01
*H–2^ds^* (12)	10.7 ± 3.3	14,375 ± 5,000	
*H–2^ss^* (12)	8.4 ± 4.5	16,933 ± 4,956	NS

^1)^Mean total IgE levels in 2 – 4 weeks after infection. Mean ± SD.

^2)^ *T. spiralis* muscle larvae were recovered 8 weeks after infection with 200 muscle larvae. Mean ± SD.

^3)^Total IgE and *T. spiralis* muscle larvae recovered.

^4)^ *T. spiralis* infected mice were immunized with 10 μg dinitrophenyl (DNP)–conjugated *T. spiralis* antigen with 2 mg Al(OH)_3_ 4 weeks after infection. The IgE response was determined by anti–DNP IgE production 1 to 3 weeks after immunization. Mice with anti–DNP IgE PCA titer higher than 160 were referred to as high responders and lower than 40 as low responders.

In addition, the involvement of the H–2 haplotype on susceptibility was evaluated in the backcross mice. The H–2 genotype of BALB/c mice is *H–2^dd^* and that of SJL/J mice is *H–2^ss^*. Therefore, the H–2 genotype of backcross mice is *H–2^ss^* or *H–2^sd^*. Approximately half of the backcross mice expressed *H–2^d^*. Comparable numbers of *T. spiralis* were recovered from *H–2^sd^* and *H–2^ss^* mice. Total IgE levels were equivalent in these *H–2^sd^* and *H–2^ss^* mice. (**Table 2**).

A similar experiment was performed in backcross mice classified as high and low IgE responders by anti–*T. spiralis* IgE PCA titers 3 weeks after infection. Total IgE levels in high IgE responders, classified on the basis of anti–*T. spiralis* IgE PCA titers > 80, were approximately twice as high as those in low IgE responders, with anti–*T. spiralis* IgE PCA titers < 40 (**Table 3**). There were 10 high IgE responder mice and 8 low IgE responders. The number of muscle larvae recovered 8 weeks after *T. spiralis* infection was significantly greater from low IgE responders than from high IgE responders. Approximately 50% of backcross mice in these experiments were high IgE responders (**Tables 2**, **3**), indicating a single gene control of IgE responsiveness.

**Table 3:** Susceptibility to *T. spiralis* in (BALB/c × SJL/J) F1 × SJL/J backcross mice classified by anti–*T. spiralis* IgE production.

Backcross mouse (n)	Total IgE ^1)^ (μg/ml)	*T. spiralis* muscle larvae recovered^2)^	P^3)^
High IgE responder (10)^4)^	6.7 ± 2.7	10,050 ± 1,541	
Low IgE responder (8)	3.4 ± 1.8	15,087 ± 5,862	<0.05

^1)^Mean total IgE levels in 2 to 4 weeks after infection. Mean ± SD.

^2)^*T. spiralis* muscle larvae were recovered 8 weeks after infection with 200 muscle larvae. Mean ± SD.

^3)^Total IgE and *T. spiralis* muscle larvae recovered.

^4)^IgE response was determined by anti–*T. spiralis* IgE production 3 weeks after infection. Mice with anti–*T. spiralis* IgE PCA titers higher than 80 were referred to as high responders and lower than 40 as low responders.

## 4 Discussion

In the present study, IgE–dependent susceptibility to *T. spiralis* was demonstrated in high IgE responder strains of mice except in the C3H/HeJ mice by comparing anti–IgE–treated mice with IgE–producing control mice. In low IgE responder SJL/J mice, anti–IgE treatment did not have a significant effect on susceptibility (**Table 1**). These results obtained with anti–IgE–treated mice agree with previous studies of genetic IgE–deficient mice such as IgE gene knockout BALB/c mice (14) and IgE–deficient SJA/9 mice (15). Moreover, these findings show that the discrepancy in IgE–dependent susceptibility to *T. spiralis* is a result of a difference in IgE responsiveness. The advantage of the protocol used in the present study with the repeated injection of anti–IgE antibodies is that IgE suppression can be induced in every strain of mice. It is not easy to establish each strain of IgE– knockout mice. Some parasites are susceptible to limited strains of mice. This protocol is applicable to these cases to analyze the effect of IgE. In contrast to IgE–suppressed mice with anti–IgE treatment, I reported the effect of high levels of IgE in mice with repeated injections of anti–hapten IgE monoclonal antibody on the protective activity against *T. spiralis* (22). The protective activity against *T. spiralis* was impaired by high levels of IgE in high IgE– responder mice but not in low IgE– responder mice, thereby, suggesting that protective activity is controlled by IgE responsiveness.

The genetic trait of IgE responsiveness also controls the susceptibility to *T. spiralis*. The present crossing experiments demonstrated that F1 mice were high IgE responders and that backcross mice were segregated into high and low IgE responder groups at a 1:1 ratio (**Figure 3**, **Tables 2**, **3**). These results are entirely consistent with our earlier findings that the phenotype of a low IgE responder is inherited as a recessive trait controlled by a single gene (4). Moreover, antigen–specific IgE antibody titers, evaluated by PCA reaction, reflected total IgE levels. High IgE responders, evaluated by anti–DNP IgE antibody and anti–*T. spiralis* IgE antibody, produced high total IgE levels. Moreover, high IgE responders produced high levels of both anti–DNP IgE antibody and anti–*T. spiralis* IgE antibody. Major characteristics of this trait are the control restricted to IgE production and the control not restricted to antigen immunization (4). It should be noted that in crossing experiments, high IgE responders always exhibited low susceptibility to *T. spiralis*, suggesting that the genetic trait of IgE responsiveness functions as a trait of susceptibility to *T. spiralis*.

To understand the control mechanism of IgE production, it is important to identify the locus of IgE responsiveness. I was unable to determine the gene locus in this work. In humans, the genetic control of total serum IgE concentration has been investigated with an antigen non–specific fashion as the IgE responsiveness in mice demonstrated in the present study. A gene controlling total serum IgE concentration in humans is linked to chromosome 5q31.1, especially to the *IL4* gene (23). Similar attempts were made in mice using crossing experiments with high and low IgE responders such as SJL/J, ASW mice, and (ASW × SJL/J) F1 × SJL/J backcross mice. Antigen non–specific control of IgE responsiveness was found in mice with repeated immunizations of several antigens. The IgE responsiveness was controlled by a single autosomal gene and low IgE response was a recessive trait. The IgE responsiveness gene was not specified and was not linked to the *IL4* gene or the *IFNG* gene. In addition, the amounts of IL–4 and interferon–γ in the culture supernatant of spleen cells from immunized mice were not correlated to IgE responsiveness (24). Cytokines are candidates for understanding the control of IgE responsiveness in antigen non–specific manner. The molecular mechanisms of IgE responsiveness provide useful information for the protective roles against parasites, as well as for the clinical treatment of allergic patients.

This study focused on *T. spiralis* and H–2 to confirm the lack of genetic linkage between IgE responsiveness and H–2 (4, 24, 25). In fact, among mice with the *H–2^s^* haplotype, SJL/J mice were low IgE responders and ASW mice were high IgE responders (**Table 1**, **Figure 3**). Between *H–2^ss^* and *H–2^sd^* backcross mice, neither total IgE levels nor susceptibility to *T. spiralis* differed significantly, suggesting H–2–independent regulation (**Table 2**). On the other hand, H–2–linked genes controlling susceptibility to *T. spiralis*, namely *Ts–1* and *Ts–2* (17), have been extensively studied. An inverse relationship between anti–*T. spiralis* IgE antibody levels and the number of *T. spiralis* larvae in mouse strains including SJL/J mice were reported to be under the control of H–2–linked and non–H–2–linked genes (26). Generally, H–2–linked genes control immune responses in an antigen–specific manner and are not specific for immunoglobulin isotypes. H–2 molecules present specific antigens and control immune responses. However, the inheritance of IgE responsiveness in the present study operates in an antigen–non–specific manner that adapts to the IgE response against helminths and ectoparasites with a complicated antigen composition.

The target of IgE–dependent protection was not specified in the present study. However, anti–*T. spiralis* IgE antibodies were detected 2 weeks after infection but not 1 week after infection (**Figure 1**), indicating that the period between 1 and 2 weeks was a starting point of IgE–dependent protection. In a previous study, I suggested that IgE antibodies play a protective role 1 to 3 weeks after infection (22). This period corresponds to all stages of *T. spiralis*. In IgE–knockout BALB/c mice, the number of muscle larvae is reportedly twice that in control BALB/c mice, and the expulsion of adult worms from the intestine is slower than that in control mice (14). Although more muscle larvae in IgE–knockout mice might reflect the expulsion of adult worms, histological observation has revealed that damaged muscle larvae are reduced in IgE–knockout mice, implying that the establishment of muscle larvae is also affected by IgE antibodies (14).

The genetic control of IgE production by an atopy gene has been investigated, and several candidate genes have been reported (27, 28). Atopy is the predisposition to allergic diseases that are harmful to humans. On the other hand, a benefit for the host is the protective role of IgE antibodies against helminth infections. Although many studies have been performed to identify the involvement of IgE antibodies in the immunity against parasitic infections including those with ectoparasites (29, 30), few studies have evaluated the genetic relationship between IgE production and susceptibility. The present study suggests that the genetic trait of IgE responsiveness functions as a trait of susceptibility to *T. spiralis*. High IgE responsiveness results in high protective activity as a dominant trait. IgE antibody is one of the factors responsible for susceptibility. In *Schistosoma* infection, IgE has protective roles and relates to pathogenesis in humans (31). IgE also participates in *Schistosoma* egg–granuloma formation in mice (32). *Schistosoma* infection is a significant issue in humans and domestic animals. The development of vaccines for the prevention of infection and inhibition of pathogenesis is required. The genetic control of IgE responsiveness may be an important factor for vaccine development.

## Data availability statement

The original contributions presented in the study are included in the article/supplementary material. Further inquiries can be directed to the corresponding author.

## Ethics statement

The animal study was reviewed and approved by the Animal Care and Use Committee of The Jikei University.

## Author contributions

The author confirms being the sole contributor of this work and has approved it for publication.
